# Biotechnology activism is dead; long live biotechnology activism! The lure and legacy of market-based food movement strategies

**DOI:** 10.1007/s10460-023-10501-y

**Published:** 2023-09-05

**Authors:** Gabriela Pechlaner

**Affiliations:** School of Culture, Media and Society, University of the Fraser Valley, 33844 King Road, Abbotsford, BC V2S 7M8 Canada

**Keywords:** Food movements, Food labelling, Third Party certification, Genetically modified organisms (GMOs), Neoliberalism

## Abstract

Scholarly debate over the transformative potential of neoliberal, market-based, food movement strategies historically contrasts those who value their potential to reform the food-system from the inside against those who argue that their use concedes the primacy of the market, creates citizen-consumers, and undermines overall movement goals. While narrow case studies have provided important amendments, the legacy of such strategies requires impacts to be evaluated both contextually and more broadly than the specific activism. This study thus conceptualizes the ‘case’ of U.S. biotechnology market activism expansively, drawing on interviews with 25 activists from diverse organizations to investigate the legacy of two food-labeling movement strategies (one public and mandatory, one private and voluntary). The results support that the legacy of market strategies extends more broadly than the immediate initiative. They also confirm that the consequences of such neoliberalized strategies are most productively assessed contextually and applied, rather than categorically—as most clearly illustrated by the counterintuitive results of the failed mandatory labeling effort. Of the two market strategies, voluntary labeling demonstrated the most problematic relationship to broader movement goals of food system transformation, in part because of the greater potential for overlapping credence claims and in part due to the risks of niche market logic.

## Introduction

Is the anti-biotechnology movement over, as one proponent of the technologies asserted (Brazeau 2018)? The biotech genie is certainly fully out of the bottle after two plus decades. Genetically modified [GM]^1^ crops now dominate the production of major U.S. food crops (e.g. in 2017, GMO adoption rates were: maize 93%; soybeans 94%; canola 100%; and sugar beets 100% (ISAAA 2018, p. 9–12) and GMOs are estimated to be in approximately 75% of U.S. processed food items (Center for Food Safety n.d.a). Corporate agribusinesses—major seed, chemical and biotechnology companies—also have power and resources on their side, and the U.S. federal government has been a steadfast proponent of the technology, facilitating its growth through a supportive regulatory regime (Pechlaner 2012).

Historically, GMO activism has kept pace seemingly undaunted, however, with the movement strategically reinventing itself after every setback, to employ a full range of strategies: traditional protests; marches; information campaigns; dramatic staging; public hearings and petitions (Kinchy et al. 2008; Schurman and Munro 2010); corporate targeting and buycotts (Bain and Dandachi 2014; Roff 2007); independent GMO testing (Bratspies 2003); local level bans (Roff 2008; Walsh Dilley 2009); and various legal challenges, such as over patent infringement (Pechlaner 2012) and litigation over the carcinogenicity of Roundup (Levin and Greenfield 2018).

Consistent, wide-spread opposition has indubitably slowed the growth of the technology in the U.S. (Schurman and Munro 2010; Schurman 2004), but concrete successes have been few. Given the dearth of effective options, activists ultimately turned their attention to neoliberal market-based strategies. While the biotechnology movement writ large is decentralized and multifaceted—a loose network of diverse groups, each with their own goals, strategies and temporary alliances (Schurman and Munro 2003)—later efforts converged onto two, overlapping, market-based strategies: (1) in the public realm, it involved attempts to gain state-level mandatory GMO labeling; (2) in the private realm, it involved efforts to establish a third party certified, voluntary standard for non-GMO content. Both approaches allowed for a practical outcome—providing consumers and activists with a means to avoid consuming GMOs—*and* transformative goals, through consumer pressure on the market. While there are significant differences between public and private strategies, these two approaches’ common use of the market to achieve their goals raise similar flags—despite the practical aspects of market strategies in GMO activism, there has been much scholarly debate over such strategies’ ability to transform the food system, and their potential to undermine the very movements they seek to advance (Alkon 2014; Friedmann and McNair 2008; Guthman 2008a, b; Harris 2009; Harrison 2008; Konefal 2013; LaForge et al. 2017; Leslie 2017; McClintock 2014; Negowetti 2020; Raynolds 2012; Sbicca 2014).

Alternative Food Movements [AFM] of all types—organic, fair trade, GMO free, etc.—increasing use of the market is borne out of frustrated efforts to otherwise challenge the social and environmental irrationalities of a neoliberalized, high-capital, production-oriented, globalized food and agriculture system. While various food movements differ on how they seek to transform the food system, depending on their particular issues of interest (e.g. reducing chemical usage, improving animal welfare), there is a significant amount of overlap in their broader goals, with most supporting a less corporate, lower input, and more socially and environmentally just and sustainable model of food production. Importantly, implicit in these goals is the desire for a food system that is more responsive to the wishes of its citizens and that attends to the many citizen values that fall outside of the current system’s profit orientation.

The AFMs who use market strategies hope to reform the system from the inside at the cost of a contradiction-riddled relationship with the market they wish to change—most significantly, such strategies have the potential to reinforce neoliberalization while simultaneously misleading consumers about their transformative capabilities (Parker et al. 2019). Scholarship on AFMs use of market strategies has been polarized, with some characterizing this use as a context-specific ‘work around’ and others arguing it undermines AFM’s ‘real’ goals, with these views variously contrasted as cooperative vs. radical, reformative vs. transformative, builder vs. warrior, and the like (Friedmann and McNair 2008; Holt-Giménez and Shattuck 2011, see also Auld 2020). More recently, there are acknowledgements of grey areas, suggesting that movements are neither fully coopted nor fully retain their transformative potential (LaForge et al. 2017; Leslie 2017; McClintock 2014; Sbicca 2014; Pechlaner 2020). The next step is thus to unpack in what ways, when, and how they retain their transformative potential.

The scholarship has furthermore largely focused on the strategies’ *direct* impact on system transformation (e.g. by reifying neoliberal values or sanctioning regulation-by-consumers) rather than their *indirect* impact on it, such as through their influence on the movements that created them. This scholarly gap is compounded by very narrow conceptualizations of the ‘movement’ in question. If we are concerned with transformative potential, however, we need to investigate the impact of market-based strategies not only on the activists that initiate them, but on the broader movement. This study thus aims to increase understanding of the wider consequences, or ‘legacy,’ of market-based GMO activism on the diverse network that comprises the biotechnology movement. Drawing on interviews with a range of GMO activists,^2^ this study investigates to what extent the turn to market strategies by some affects the transformative potential of the movement more broadly, and it considers what further insights this can provide into market strategies’ potential for food system transformation.

The next section contextualizes biotechnology in the neoliberalization of the agri-food system and outlines the debates over market strategies’ transformative potential. This is followed by the methods and a brief background of the two GMO labeling efforts—a state-level mandatory GMO label, and a voluntary, private non-GMO standard. The results demonstrate that the ‘legacy’ of such strategies *can* extend broadly and that neoliberalization impacts are most productively assessed contextually rather than categorically, as is most clearly illustrated by the counterintuitive results of the failed mandatory labeling effort. Of the two strategies, voluntary labeling had the most problematic relationship to broader movement goals.

### Biotechnology activism in theoretical context

While certainly very publicized, the fight over GMOs is just one of many food system battles. Indeed, if we add up the many food-related issues of contention, the level of public distrust and social agitation suggests the food system is woefully under-performing on many social, health, and environmental concerns. It is, however, a neoliberalized food system that is highly effective at meeting capital accumulation goals. Despite the concept’s definitional ambiguity, neoliberalization remains an important signifier of processes that occur in both the regulatory and cultural environment. With respect to the regulation of agriculture and food, neoliberalism predominantly occurs in conjunction with state-led globalization imperatives that privilege free trade for international agribusiness (McMichael 2009). It is not synonymous with deregulation, as regulatory imperatives include important state interventions to facilitate privatization and market liberalization (Pechlaner and Otero 2010), nor are neoliberal processes limited to economic and regulatory fields. Rather, in the manner of Foucauldian governmentality, a neoliberal society is remodelled after the market (Byrne 2017; LaForge et al. 2017); neoliberal ideologies infiltrate and reshape social relations, dominate over non-market values, and help create populations of citizen-consumers (Guthman 2008a; LaForge et al. 2017).

Holt-Gimenez (2019) consequently argues that the capitalist food system is working exactly as expected: “It overproduces, it concentrates power in capital in the hands of a few, and it leaves us with all the externalities” (2019, p. 9). Ioris (2015) similarly characterizes neoliberalized food production in the global south as “less about food, nutrition and well-being” than “short-term capital accumulation” (2015, p. 2). While issues differ regionally, there is no shortage of negative impacts NOR of food movements attempting to address them. As Scott (2009) cogently conceptualizes, such undesirable outcomes are not about Smith’s ‘invisible hand,’ but about the visible hand of political agency.

Not to overstate the case, this ‘agency’ is inextricably tied to political-economic context, as is most notable in the profound contrast between the comprehensive precautionary approach to GMO regulation evidenced in the European Union and the lax and fragmented regulatory approach in the U.S. The European outcome was affected by a greater cultural sensitivity to food, heightened by food safety incidents such as the mid-1990s BSE outbreak (leading the European public to become critical of the technology earlier than the American public (Bernauer and Meins 2003; Kurzer and Cooper 2007), a “broad and influential anti-GMO coalition” (Bernauer and Meins 2003, p. 653), and to challenges that regulating the controversial technology posed to member state consensus in the fledgling union (Seifert 2008; Pechlaner 2012). Responding to such bottom-up pressures, in 1997 several EU member states’ use of a safeguard clause (related to health and environmental risks) in order to ban EU-approved GM crops from their territories triggered a de facto moratorium pending new regulations. The resulting comprehensive labelling and traceability regulations (enacted in 2004) required “detailed information gathering, risk assessment, and tracking and monitoring procedures” and emphasized transparency, long-term assessment and public consultation (Pechlaner 2012, p. 66). This political economic context has continued to differently influence the EU regulatory framework, as more recently illustrated in 2015 allowances for member states to “restrict or prohibit” GM crop cultivation for reasons “other than risk-related criteria” (Eriksson et al. 2020, p. 231; Salvi 2016).

Despite this apparently successful Polanyian (2001 [1944]) societal self-protective measure influencing EU regulations, Carroll (2016) argues that any such view “is insufficiently conscious of the way power relations torque the direction of self-protection” (2016, p. 20). Drawing on case studies in the EU and Australia, Carroll illustrates how even social and environmental protective measures can be transformed to “cohere with the project of neoliberal hegemony” (19), demonstrating with respect to the EU, for example, how business and consumer actors were preferenced over southern farmers in regulatory outcomes. Scott’s ‘visible hand’ thus continues to shape market frameworks in ways that are disadvantageous to social and environmental movements, albeit this disadvantage has been far greater for U.S. GMO movements, where a centralized authority could more productively pursue its development of an American biotechnology empire (Pechlaner 2012).

While the arena for US biotechnology resistance is unfavourable, neoliberalism is not a fait accompli but a process (Andree et al. 2015; Guthman 2008a; Kinchy et al. 2008; McClintock 2014), and resistance has affected its implementation. This is most notably evidenced in the defeat of Roundup Ready wheat (Eaton 2015) and GM flax (Camille and Smyth 2012), and the banning of rBST in Canada, the EU and other countries (MacMillan 2003). Yet, despite substantial effort and some promising wins, activists have failed to achieve substantive change in GMO regulation. In part, this is because of the technologies’ import in a neoliberalized food system—aided by a highly supportive regulatory environment, they promote capital intensive agriculture, facilitate accumulation, and solidify the primacy of agribusiness multinationals (Kinchy et al. 2008; Torrado 2016). This failure has also been facilitated by discourses linking neoliberalism with ‘scientism’ and by very narrowly defined risk assessments (Kinchy et al. 2008; Kinchy 2012); rather than allow social debate about capital concentration, risk tolerance, etc., those with concerns are effectively discounted as unscientific (e.g. Fischer et al. 2015; Hilbeck et al. 2015). Discounting GMO concerns this way, neglects the public’s reasonable unease over relying on their food system to attend to issues outside of capital accumulation, however. Messer et al. (2017), for example, suggest that mandatory food labels (such as for GMOs) are detrimental to consumers because market avoidance could reduce product choice and, in the long-term, “[curtail] the historical steady rate of progress in food production” (2017, p. 419)—neglecting that it is to this very ‘progress’ that consumers have become wary.

The GMO activists’ turn to market-based strategies in the face of regulatory impasse reflected similar efforts made in other movements (Auld 2020; Challies 2012; Konefal 2013; Lyon 2020; Negowetti 2020). Early celebration over such strategies’ ability to transform the food system was soon met with concern over their potential to reinforce neoliberal processes, however. Guthman (2007, 2008a, b) offered the most profound challenge, detailing the ways in which market-based activism fails to “[name] and [address] actually existing neoliberalizations of the food system” (2008a, p. 1180). The main concern is that market strategies ultimately reify neoliberal processes—they support the primacy of the market as the organizing feature of social life and locus of regulatory control, emphasize entrepreneurialism, and responsibilize individuals to care for formerly state-regulated issues. Market strategies thus depoliticize problems by shifting their resolution to individual purchasing decisions (Guthman 2008a; Roff 2007). For example, in an investigation of sustainable seafood, Konefal (2013) argued that the strategies conveyed that “the market is the appropriate mechanism for environmental governance” (2013, p. 339). By partnering with the system they want to change, activists implicitly condone it—and concede defeat. Challies (2012) similarly argues that activists defeat their own goals with market strategies, as these strategies obscure realities, represent “corporate actors as ethical and responsible,” and “[create illusions] of progress,” perpetuating the status quo (2012, p. 189).

Moreover, such neoliberal governmentalities can affect activists’ abilities to think outside of “the neoliberal present” (Guthman 2008b, p. 1251), in a way that compromises even radical visions (Alkon and Mares 2012). This strategic lapse explains activists’ contradictory reproduction of neoliberal forms and spaces of governance while opposing “neoliberalism writ large” (Guthman 2008a, p. 1172). An example of neoliberal ideas colonizing resistances this way can be found in efforts to court consumers during the GMO-labeling ballot initiatives, where pro-GMO neoliberal arguments regarding “food safety, cost, and choice” were countered by activists’ equally neoliberal arguments for consumers’ ‘right to know’ (Bain and Dandachi 2014, p. 9464).

Market-based strategies’ politicized consumption is often facilitated by third party certified standards which can exacerbate challenges (Brown and Getz 2008, p. 15). Little and Lucier (2017), for example, found “considerable potential for these standards to be compromised and transformed into a vehicle for corporate legitimation and economization” (2017, p. 212). In a rare study of the private GMO labeling initiative investigated here, the Non-GMO Project, Roff (2009) documented how alliances with industry partners altered the activists’ agenda and caused it to lose its oppositional goals. While the Project’s non-GMO standard was created as a tool to avoid, and consequently eliminate, GMOs, efforts to broaden participation led to changes—e.g. label premiums as incentives for manufacturers (which increased the cost for consumers), higher non-GMO assessment fees (which priced out small manufacturers), and a shifting threshold of GMO tolerance—whereby the project ultimately created “a premium parallel market for non-GMO foods” (2009, p. 353) rather than a vehicle for transformative change.

In sum, scholarship would seem to dissuade against the use of market-based activism for food system transformation. However, just as neoliberalizations are “contingent, uneven and contested” (Sbicca and Myers 2017, p. 3), so too are its resistances, and more recent studies suggest qualifications to the neoliberal critique. Increasingly, scholarship debating the neoliberalization of market strategies focuses less on a black versus white polarity, and more on the grey areas, the mixed results, and the scale of transformative potential. McClintock (2014), for example, in a study of urban agriculture, argued that binary views of neoliberalization are oversimplifications; rather, neoliberalizations “may exemplify *both* a form of actually existing neoliberalism *and* a simultaneous radical counter-movement” (2014, p. 148, italics in original). Essentially, McClintock contended, “contradictory processes of capitalism both *create opportunities for* urban agriculture and *impose obstacles* to its expansion” (148, italics in original). This perspective finds convincing support. In investigating ferias francas *(*an Argentinian type of farmer’s market), for example, Leslie (2017) similarly argued that even while using the market, the ferias nonetheless acted to contest neoliberalism in many aspects of their operation. Andree et al.’s (2015) investigation of community-based food initiatives in Canada found that while activists tried “to create favourable conditions for small alternatives” rather than challenge the state, they also simultaneously worked on systemic change through “tactical and multi-faceted” entrepreneurial engagement (2015, p. 1467-8). This suggests the benefit of a more nuanced approach to investigating neoliberalization in market-based activism, such as what might be revealed by a broader, more contextual analysis.

There is support for this idea that neoliberal strategies’ transformative potential is inextricably linked to context. For example, in Sbicca’s (2014) study of food provisioning/justice organizations, Orlando Food Not Bombs effectively acted out a “neoliberal response” through feeding people, but fought to do so publicly in order to “visually display the failures of neoliberalization” and the “gap between rich and poor” (2014, p. 830). Konefal (2013) similarly argued that while market strategies have serious limitations and can pose risks to movement goals, they should not be outright abandoned as they can still “slow environmental degradation and have localized affects” (2012, p. 348). Responsiveness to context thus appears central to effective resistance. Importantly, Harrison (2008) concluded that the danger of neoliberalization is less about the “carrots” (market-based activism) than about the lack of “regulatory ‘sticks’”—and the danger is gravest when market strategies are “severed from the broader fights for responsible and fair government protection” (2008, p. 1211). This is arguably an essential distinction. In short, as Leslie (2017) argued, it is still very much “an open question as to which neoliberal tools can be used to successfully challenge neoliberalism” and which are counter-productive to movement goals (2017, p. 739)—to which we could further qualify in which ways and in what context?

While the above scholars provide insight into the complexities of social movements’ use of market-based strategies, their investigations are primarily limited to the specific organizations that initiated the strategies rather than the broader movement. This gap mirrors shortcomings in social movement scholarship, which is robust regarding the tools and opportunities of mobilization but far weaker on its outcomes (Guigni 2008), particularly with respect to the social and cultural impacts that fall outside of legal and policy realms but that have great importance for real world change—although advances on these difficult-to-measure outcomes are slowly increasing (e.g. Chiarello 2018; Polletta 2012). Similarly, in this investigation, market strategies’ impact on transformative potential needs to be assessed from their *direct* effect as well as from their *indirect* effect on the broader movement itself, which, in turn, can affect the potential for future food system transformation. For example, while Roff’s (2009) study of the Non-GMO Project’s private labelling strategy illuminated its impact on its initiators’ goals, it was silent about its impact on the broader GMO movement. Activisms’ consequences can reach far beyond its initiators, however, particularly in a movement as large, heterogeneous, and networked as the GMO movement. Investigating how market strategies affect this broader movement in this way is an important addition to understanding their legacy and their impact on the potential for food system transformation.

## Methods

In-depth, qualitative data was gathered through a case study approach in order to investigate this ‘case’ of market-based GMO activism (Yin 2018). Many activists were keenly attentive to the state-level mandatory labelling efforts (and some were later involved with the draft federal labeling regulations), while the voluntary, Non-GMO Project [NGMOP] private standard was widely publicized in response to repeated failures to gain mandatory regulations.

For this case study, the broader GMO movement was defined by participation in GMO-related activism. Interviewees were purposively sampled for the extent of their (or their organization’s) involvement in GMO activism. Subjects were primarily identified through online searches of news articles, GMO-specific websites and Facebook pages, and content pages of related established organizations (e.g. environmental, food/agriculture, consumer), with additional subjects sought through snowball sampling. Organizational participants included GMO-specific organizations that emerged directly from GMO mobilization efforts, as well as non-GMO specific organizations (e.g. environmental, consumer, health, and farm or rural related organizations) that adopted GMOs as an organizational interest. Participants were primarily organization leaders, leaders with respect to GMO campaigns for broader themed organizations, or otherwise important players in GMO activism. Twenty-five interviews were conducted with activists from 22 different organizations. Overall, the interviewees effectively captured the diversity of biotechnology activism and organizations, with 1–2 omissions.^3^

Interviews were conducted by telephone, between September to December 2018, prior to the release of the highly problematic final federal labeling rules. Interviews were semi-structured, with questions modified depending on the interviewee’s activism and involvement with market-based strategies. Questions were designed to help ascertain how the labelling strategies affected the broader movement and its transformative potential, paying attention to the concerns scholars have raised to date over activists’ use of such strategies. While assessing movement impacts is difficult, it can be indicated by professional impacts, (e.g. on organizational goals and strategies), movement coherence, and personal impacts on activists, such as the extent and direction of their ongoing activism.

With respect to the first two, for example, activists were asked about their relationship to the labelling strategies, and the extent to which these actions aligned with or frustrated their own professional goals; to what extent did these strategies divide the movement and its resources, causing tensions and potentially derailing more transformative efforts (versus foster alliances, unity and movement coherence)? An important, related, component regards how sensitized activists were to the issues of concerns raised by scholars over such strategies’ undermining potential—e.g. the abdication of regulatory authority, the responsibilization of consumers—as dissention over such concerns could affect movement unity. Another important indicator relates to these strategies’ potential impact on the radicalness of activists’ goals and strategies, such their ability to think outside Guthman’s (2008b) neoliberal present; are activists able to be simultaneously radical (McClintock 2014) and retain focus on broader fights for government protection (Harrison 2008), or has their imagination been captured by the market as ‘solution’?

Activists were additionally asked their perspective of the strategies, including their experience with any benefits or drawbacks. This can provide evidence of the direct impact of the strategies’ transformative potential—relating to scholarly concerns regarding cooptation of values, etc.—and can additionally indicate whether and how strategy outcomes personally impacted activists, for example by empowering or disillusioning them. Further evidence of the personal impact on activists that would indicate movement strength is the extent they remain motivated and engaged as activists in related work.

Interviews were transcribed and thematically analyzed with the aid of NVivo data analysis software. While activists could feel politically compelled to emphasize movement successes, careful questioning about activists’ relationship to market-based strategies and these strategies’ influence on their organizations and their activism aimed to promote more genuine insight into their impact.

## Labeling

While there had been earlier GMO labelling attempts—notably with respect to rBST in milk—labelling strategies gained greater traction around 2010 (Velardi and Selfa 2021) when both the private, voluntary, Non-GMO Project labeling effort and the state-level, mandatory GMO labeling efforts became lightning rods for activism in the broader GMO movement. With respect to mandatory labeling, federal regulatory indifference to sustained public concern fueled numerous state-level attempts to pass GMO labeling. Federal assurances that GMO foods were no different than conventional ones notwithstanding, polls indicated sustained, strong public support for labeling in the U.S.; in 2013 the New York Times found 93% support (Kopinki 2013); in 2014 Consumer Reports found 92% support (Consumer Reports National Research Center 2014); and in 2015, the Mellman Groups Inc. found 89% support (Centre for Food Safety [CFS] n.d.b). Contrasting federal support for GMOs, many state governments expressed caution, finding that GMOs “potentially pose risks to health, safety, agriculture and the environment, necessitating legislation involving [their] labeling” (Nat 2016, p. 207). State-level efforts for mandatory GMO labeling took off early in Oregon in 2002, followed by a succession of attempts a decade later. Strategically, widespread state-level GMO labelling—carefully crafted so as not to conflict with existing federal laws—could force manufacturers to adopt these labels nationally for ease of marketing, thus facilitating labelling even where it was not passed. By 2014, 54 labeling bills had been introduced in 26 states (CFS 2014).

Food industry opposition to labelling was fulsome and well-resourced. In four key ballot initiatives,^4^ for example—California (2012), Colorado (2014), Oregon (2014) and Washington (2013)—opponents of labeling outspent proponents four-fold ($101.1 versus 25.9 million) (Bain and Dandachi 2014, p. 9465). It is a testament to the support for labeling, that many battles were tight despite the resource imbalance. In the above four initiatives, labelling’s proponents received 48.6% (California), 34.5% (Colorado), 49.9% (Oregon) and 48.9% (Washington) (Ballotpedia n.d.a.; n.d.b; n.b.d.; n.d.e.). Ultimately, Connecticut (2013) and Maine (2014) became the first states to successfully pass labeling legislation, although both had trigger mechanisms that required a threshold of similar legislation for enactment.^5^

Finally, in 2014, Vermont became the first state to successfully pass unqualified GMO labeling legislation, with a 2016 implementation date. In the face of this success, the federal government moved to enact legislation that would pre-empt it.^6^ After two attempts, it succeeded, replacing Vermont’s law (and nullifying other state actions) with an impending federal GMO labeling law (the National Bioengineered Food Disclosure Standard [NBFDS]). Unlike the comprehensive EU labelling legislation, most activists argued that the NBFDS deliberately obstructed their goals. In response to the final regulations released in December 2018, for example, the Center for Food Safety stated, “the USDA has betrayed the public trust by denying Americans the right to know how their food is produced” (CFS 2018), the Organic Trade Association [OTA] called them “deeply disappointing” (OTA n.d.), the Environmental Working Group stated they are “denying Americans basic information,” and Consumer Reports found they “[fail] to give consumers the information they deserve” (Watson 2018).

Overlapping these efforts, and in the same spirit of diminishing strategic alternatives, some frustrated activists sought to achieve their goals through private labeling. As Bain and Dandachi note (2014), the high-resource, high-stakes, state-level battles “energized the anti-GMO movement, garnered significant media attention, fuelled a national debate, and raised public awareness about GMOs” (2014, p. 9469), which helped set the stage for private labeling efforts. A voluntary, non-GMO label would provide consumers the means to avoid GMO foods (although with less coverage than mandatory labels) and could still allow them to apply pressure for GMOs’ ultimate removal, thus it was still transformational in intent.^7^

In 2002, the Natural Grocery Company (Berkeley, CA) responded to customer concerns by cataloging products for their GMO risk, spurring a “People Want to Know” campaign for greater retail support and eventually expanding into Canada in alliance with the Big Carrot Natural Food Market (Roff 2009: 356). The Big Carrot had similarly engaged with GMOs for their customers and both stores worked to increase retail participation but struggled with the lack of consistent non-GMO standards and “uneven” manufacturer response (Roff 2009, p. 357). They ultimately partnered with a firm engaged in non-GMO certification for international markets and established a standardized verification process, rebranding as the Non-GMO Project (357). Products bearing the NGMOP labels reached market in 2010, and rapidly expanded to currently representing over 50,000 products and $26 billion in sales (NGMOP n.d.). While the NGMOP is still the most dominant non-GMO certification, by 2017 there were already six more (Broaddus 2017), and other companies assert non-GMO status without independent verification, leading many to call for federal involvement to regulate consistency.

## Results

### State-level labelling

The most straight-forward indicator of market strategies’ effect on the broader movement comes from activists’ perception of its impact on their goals. Activists had a variety of motivations for their GMO activism: environmental, health and safety, corporate concentration, and small farmer sustainability, among others. While their motivational objections to the technologies varied, most additionally objected to what they perceived as the ‘real’ purpose of GMOs—profit and power. Respondents frequently made statements such as, “There’s one reason, and one reason only, for agricultural biotech, and that’s control” [10], and nearly all had a very low opinion of the government’s willingness to regulate GMOs and felt that it protected industry over citizens. Consistently, the functioning of the regulatory system for GMOs was most generously characterized as ‘weak,’ and less generously, and far more commonly, as symbolic of a democratic crisis. The federal government and its relevant agencies were variously called: sellouts, corrupted, rubber-stamp regulators, untrustworthy, biased, co-opted, and worse. They were accused of being under corporate influence or otherwise in collusion, resulting in pre-determined political outcomes and deafness to the will of the people. A common sentiment amongst activists was that “[I]n the US, we let the companies dictate the regulations” [17].

Motivational and organizational diversity aside, the broader biotechnology movement united behind mandatory labeling. Almost all activists were theoretically supportive of the state-level labeling efforts, and most were at least tangentially involved, even in mobilizations outside their home state. Participation ranged from full-time activism to support roles (e.g. testifying at hearings, consulting, posting updates and events on their websites, etc.). A key reason for this unity was the ineffectiveness of prior strategies; mandatory labeling was largely viewed as a pragmatic decision in a context of diminishing effective alternatives and substantial corporate and federal opposition. Pragmatism notwithstanding, activists overall still demonstrated significant faith in the power of the market *when* consumers have sufficient information. For example:

I believe in real markets. We don’t have real markets now. [...]Why is a corporation’s right to create a product superseded by a person’s right to know what’s in it and not buy it? [6]

Within this unified pursuit of mandatory labeling, activists were fairly divided between those who felt that consumer choice through labeling was an end in itself, versus those who sought labelling as a means to an end (banning GMOs). With respect to the latter, for example:

…[S]o the end run was to label them. If labeling was enacted, it would be a de facto ban because consumer pressure would probably mean that the majority of consumer-packaged food companies would not want to have their product identified with the technology. [1]

In either case, consumer choice could have real consequences for the technology, and transformative potential for the food system.

Importantly, despite their often-tremendous resistance efforts, a number of respondents were not actually opposed to GMOs. They could envision favourable GMO applications, but felt they had no choice but to support the market strategy in the context of poor regulatory oversight. As one interviewee whose organization worked extensively on labeling put it:

[The regulatory system is] designed as a PR initiative, essentially, to get people to believe that these crops have the government stamp of approval; therefore, they’re fine, they’re safe, they’re wonderful. [...] So we feel that if there were a meaningful regulatory system, a lot of these crops wouldn’t be approved. We don’t foreclose on the idea that there could be a useful and nonhazardous GMO. [20]

Or another:

We’re asking for solid science that isn’t done by the corporations that are benefitting from their patents. [...] If there is a trait down the line that works and is safe and we are 100% sure that it’s okay, then whatever. [6]

The unanimity of disparagement of the state as regulatory protector was only heightened by federal preemption of the first state-level labeling law—which occurred within weeks of its 2016 implementation. Draft federal labeling rules were subsequently released for public comment in 2018. The details of their perceived failings are covered elsewhere (Pechlaner 2020), but they have been widely criticized for impeding consumers’ access to information (e.g. through providing a QR code or phone number for information instead of on-package labeling), biased (sunny-faced) logos, unfamiliar abbreviations (BE for bio-engineered, instead of GMO), and definitional issues that left many uncertainties—notably, whether new gene editing technologies would be included under the definition of GMO. Similarly, while the interviewees here were geographically, philosophically, and tactically diverse, few believed the proposed rules had any purpose other than to thwart them. The below responses are typical:

I mean it’s all set up to have virtually no impact, right? [18]

It’s all smoke and mirrors, because they cannot tell the truth and have a presence in the marketplace. [19]

﻿Although some hoped for the possibility of improvements to the regulations, even they frequently offered qualifications, such as the following: “You can [put lipstick on a pig], but it’s still a pig” [7].

The chronology reveals a laddering effect of activists’ negative attitude towards the state: the lack of trust in GMO regulation fostered opposition; poor state-responsiveness amplified opposition, increasingly focused on state-level labeling; the resulting, much-maligned, federal preemption reinforced activists’ perception of regulatory failure. In *this specific context*, theoretical concerns that market strategies facilitate the transfer of regulatory authority from the state to the consumer are rendered moot, as the state was already perceived to have vacated that role.

Despite the high level of support for labeling, activists were not wholly uncritical. Although expressions of comfort with the primacy of the market and consumers’ right to choose prevailed, this ‘comfort’ was often likely a carefully honed political position designed to maximize public support. Tellingly, many laced their support with qualifications. A limited number of these reflected theoretical concerns over neoliberalization. For example, on the assumption that non-GMO verified food would cost more, some activists raised the class-barrier issue, whereby labeling would only help those with the “most discretionary spending” [17]. Another concern activists raised related to reifying the ‘neoliberal citizen,’ whereby responsibilized consumers believed their only democratic obligation was informed consumption. For example:

...[W]e hear people say, ‘Oh we know; I go to the Farmers Market, I’m good, I did my thing.’ And it’s like, ‘yeah, you’re not done yet. Like, I’m going to need you to call your Senator, or any elected official, and say the word ‘food,’ right?’ [2]

Guthman’s concern over activists’ inability to think outside “the neoliberal present” (2008b, p. 1251) in their use of market strategies seems better applied here to the public they are trying to engage.

Only one interviewee actually opposed market strategies, in affinity to such theoretical concerns, arguing that they led to dead ends (e.g. quibbles over label wording), and distracted from food system transformation [16]. Countering the pragmatic support for labeling, this activist argued: “‘Nothing’ is better than ‘better than nothing’—you didn’t waste resources, you didn’t waste energy, and you didn’t lie to your people” [16]. This position was exceptional, however.

More commonly, even two years after preemption activists remained theoretically supportive of labeling, qualified by the practicalities of its ability to meet their goals. For example, in the high-capital, highly political, agricultural biotechnology battle, the potential for disjuncture between the ‘letter’ and the ‘spirit’ of any labeling rule was frequently noted, as the following view of the federal preemption illustrates: “the concern was, and was born out, that it would be, actually, a fake labeling bill” [8]. Activists were similarly attentive to other application practicalities, such as the likelihood of cooptation, low consumer awareness, and heightened confusion over multiple (often over-lapping) labels and standards. The burden on consumers to navigate labeling complexities was frequently noted, although the problem was attributed less to consumer failures than to a food system that permitted deliberate obfuscation in pursuit of profit. For example:

[Y]ou see it on pork products here. You see ‘no hormones administered,’ and it’s like, ‘oh, great; it’s illegal to give hormones to a pig, so it’s not really worth the ink...’ [2]

Application concerns aside, and despite the ultimate federal preemption, evidence suggests that in this regulatory context the state-level mandatory labelling initiatives did not threaten the broader movement’s more transformative goals; activists had no illusions about the limitations of the strategy, they did not willingly cede regulatory control to the market but remained theoretically committed (in spite of the highly resistant context), and those with more radical intentions retained them despite the ‘solution’ that labelling could provide.

### The Non-GMO project

If the drive for mandatory state-level GMO labeling can be characterized as a pragmatic fallback position, then the initiative to establish a private, third party certified, non-GMO standard was fallback to the fallback. Interviews occurred at a time most likely to reflect positively on the NGMOP given the recent release of the unfavourable draft federal labeling rules. The vast majority of respondents were indeed supportive, viewing it as a work-around for their goals: it was a “pragmatic thing” [18], that “filled a void” [11], and was a “stop-gap measure” [24] for those who wanted to avoid GMO foods. As one activist put it: “I’m very supportive […] because there is no mandatory labeling” [23]. Despite the favourable context, there were far more concerns expressed over voluntary labels in general, and the NGMOP in particular, than over mandatory labeling, and a general consensus that the NGMOP was an “imperfect solution” [2].

Activists frequently referenced organic production in interviews as its high profitability and growing market share had attracted corporate interest, raising important issues for food movements. Under corporate pressure, U.S. organic standards had faced repeated reinterpretation (e.g. regarding pasture rules and hydroponics), rendering a heretofore holistic vision designed to nurture people, animals and soil into one that maximized production volume and profit. This history was forefront for activists, who often drew on it to note that even initially strong standards require constant vigilance. In this sense, activists were cognizant of important risks inherent to market strategies but found them warranted in an aggressively resistant regulatory context.

At the same time, structural specificities of the NGMOP triggered important problems. The NGMOP was initially conceived as an ‘add on’ label to organics. While organic production ensures a *process* (farmers don’t use GMOs in production), contamination possibilities led NGMOP activists to ultimately choose *end-product testing* (which certifies for GMO absence). This difference triggered two main concerns: (1) it confused the public and ‘devalued’ the organic label; and (2) by certifying GMO absence, the NGMOP created a false market in credence claims.^8^ Both contravened broader food movement goals.

With respect to the first point, consumers were often unclear on the difference between organics and the NGMOP. The groundswell around state-level labeling helped the NGMOP gain market prominence through a public sensitized to the issue of GMOs but less clear on the complexities of ‘process’ versus ‘end-product’ labeling. The NGMOP increasingly became viewed as either synonymous with organics or as the *only* non-GMO option. This competition negatively affected the organic market—without providing the many food-system benefits that organics could (e.g. reduced synthetic chemicals). The result was tantamount to venerating pre-organic agricultural practices, which set the broader food movement back, frustrating many. As one activist lamented, what was currently being celebrated as ‘GMO-free’ had previously been disparaged as ‘chemical food.’

The NGMOP also affected broader food movement goals regarding the support of small scale production. In contrast to mandatory GMO labeling efforts (which would label GMO ‘presence’), the voluntary NGMOP standard (which labels GMO ‘absence’) burdens those who don’t use GMOs with the time and expense of verifying it. This can be prohibitively expensive for small-scale farmers, who faced pressure to double certify in the context of consumer confusion over organics’ GMO status. As one respondent noted:

We know a lot of folks in the organic industry who have taken 30 to 40 years to build that label, and people now don’t know it’s non-GMO; so you’re seeing a [NGMOP] label and expense on top of the organic certification…. [2]

The NGMOP was not only a double-edged sword for these broader food movement goals, but it problematically also created a market independent of them. Products could be marketed as NGMOP verified where no GMO equivalent existed, capturing price premiums without food system benefits. This again frustrated many activists. For example, the following activist contrasted the resulting situation against the NGMOP’s original proposal of an organic ‘add-on’ label:

It would’ve been a benefit to society and a benefit to the planet, and now, you’ve got these fraudsters—oatmeal that is non-GMO. Well, they haven’t introduced any oats that are GMO… [10]

Or another:

...I do worry a little bit when you see Non-GMO Project on olive oil and there [are] no GMO olives. […] [T]hat creates a little arms race, and all their competitors on the shelf have to put the Non-GMO Project label on their olive oil so it’s not implied that they are GMO when that doesn’t exist. [2]

A number of activists expressed apprehensions that the label could thus act more to serve the market than to foster system transformation.

While a few were impassioned about these problems, the vast majority with misgivings nonetheless remained supportive of the initiative and its activists. As one who knew those involved in the NGMOP, reflected:

It’s not about getting rid of the organic label. It was supposed to be an add-on to the organic label. Unfortunately, you can never control where things go. [6]

﻿In this context, it appears the only thing worse for food system transformation than having a voluntary, third party certified, absence standard for GMOs is not having it. Despite its drawbacks, certification helps ensure consumers’ purchase intentions are met—in contrast to the growing, unregulated, non-GMO market claims—and consequently many valued it as a “necessary evil” [21]. For example:

﻿Certification has its set of issues and can be gamed. But it’s way, way, way, way better than unverified claims, which is just a complete greenwashing. [21]

### The legacy of labeling

An important marker of the legacy of market strategies is the subsequent state of the movement as reflected by its unity, activists’ ongoing motivation, and their assessment of the trajectory of their efforts. Although GMO activists were regionally and organizationally diverse, dwindling opportunities for effective resistance, extreme regulatory resistance, and deep public support fostered an abnormally high level of convergence of support for the two strategies. This greatly heightened cross-organization networking and alliances, as many consulted with or otherwise offered high-level assistance towards their joint goal. A tremendous commonality of purpose, fuelled by broad grassroots and consumer support, heightened a lasting sense of empowerment for many. For example:

It was professionally really gratifying to be part of helping thousands and thousands of people to get on the streets and hand out leaflets and be part of a political campaign, when so many of them had never done anything like that before. [7]

This sense of purpose and unity persisted, despite the outcome. Nonetheless, post-preemption discouragement over their work being ‘wiped away’ was also a major theme for state-level labeling activists, with many finding the outcome “defeating and frustrating” [17]. Such a widespread sentiment could have repercussions for the broader movement, costing it future activists. This impact was often noted, for example:

You know, we do worry that that was people’s first experience in food activism. It wasn’t a great one.... [2]

While the mandatory labeling strategy did drain resources and dishearten some, this was not specific to the market nature of the campaign. Importantly, negative impacts were firmly attributed to the federal government’s undemocratic response rather than to the strategy itself. Less than a handful of activists expressed any negative personal impacts from their involvement and most of these were qualified. For example, one interviewee who had left activism for over a year after preemption described their return as ‘hopeful,’ as there was “so much more awareness than when [they] started” [#25]. Only one interviewee reversed their opinion on the value of their (extensive) involvement in state-level labeling, which they retrospectively considered a distraction from the food system`s “real problems”:

...[G]etting sidetracked into whether or not something is GMO is just one more thing. You can have great, non-GMO white sugar: ‘Oh, it must be healthy!’ [12]

This interviewee still engaged in activism but avoided food issues and labelling as a means to create change, rejecting the performative aspect of value-based consumption. This turning away was an anomaly, however, and the vast majority remained primarily positive about the labeling efforts and were undaunted in their activism. All the interviewees were still engaged in some form of activism. Most continued in food activism, and a small subset of those remained involved with GMOs. The multi-year delay from preemption to final federal rules had a palpable impact on GMO-specific activism—while most activists expected the worse, they were in a holding pattern until the rules were finalized. Those working on GMOs necessarily shifted from grassroots organizing to policy work, such as lobbying to strengthen the upcoming rules. For example:

This is a weak law, it’s not good, but it’s here now so we’ve got to do the best we can to try to make it as strong as possible. [20]

Definitional issues, for example, could determine whether the regulations included new gene editing technologies (e.g. CRISPR). Excluding these technologies—which could ultimately replace traditional genetic modification in crop development—could render meaningless what little GMO labelling was provided.

For the few not specifically focused on labeling but still involved with GMOs, activism varied. As one activist who shifted to supporting litigation over the carcinogenicity of Monsanto’s Roundup (Levin and Greenfield 2018) characterized it, cancer litigation was the next “awareness point” [17]. Others noted they “have to pick,” such as one small organization who stated that while labeling was not on their agenda, they would reconsider if requested by partner organizations: “We’ll throw what we have behind it, of course, because most of our folks still would like to see real GMO labeling.” [24]. As another observed: “…you just kind of fight where you can, and where you have leverage” [21]. While restrained, many still expressed hope and felt that GMO resistance was, so to speak, on “simmer” [24]. Thus, importantly, activists were no less motivated but necessarily had to redirect their efforts—not out of disillusion with labelling strategies, but for simple practicalities of maximizing their impact.

Tellingly, those who moved on from GMO activism (in whole or in part) frequently redirected their efforts to democratic reform. Over a third were involved in such activities, for example, by supporting relevant political candidates; strengthening community rights; ranked choice voting; campaign finance reform; and other forms of “getting big money out of politics” [5]. This indication of discontent with their activism’s outcome again lay more in the perceived failure of political representation than in the market strategy.

Counter-intuitively, despite acknowledgements of the dampening of GMO-related activism, activists were modestly optimistic about the movement’s overall health and expressed optimism and perseverance, such as: “kind of like most movements: you lose, lose, lose, and then you win” [21]. Or, in response to federal preemption: “Every time historically people have tried to muddy the waters or deny consumers that right to know, it hasn’t worked out well” [13]. Similarly, another noted that while labeling had “been dealt a heavy blow,” they remained optimistic on account of the recent court victory over Roundup’s^9^ carcinogenicity [23]. An important, related, theme was the conceptualization of GMO-labeling as one battle in a larger war—one “step” in the broader struggle towards “a new food system” [2]. The following illustrates this prevalent theme:

...I think biotech is part of that kind of rejection of industrial agriculture and overuse of pesticides. I think it’s all part of the same movement for a lot of folks…. [20]

The battle over GMOs resonated with a public already uneasy about the industrial food system and ready for change, and activists frequently cast themselves as facilitating this larger project’s momentum. As one activist articulated, they aimed to help people see “the dangers of not only GMOs in their food, but the whole [centralized, chemical-intensive] change in agriculture” [23]. As another argued, American culture now had “the seeds” of many contemporary food movements that were not even conceivable 20 years prior [18]. Associated with this, increasing consumer awareness was central to many activists’ views of the value of the mandatory labeling initiative:

[Labels aren’t], in themselves, like consumer education, or whatever. They are at the point of sale, but it’s, in a way, the political fights that accomplish them are what really generates the debates and the awareness. [21]

In this sense, activists did not perceive the labeling effort as a ‘loss,’ despite the disappointing outcome, but as a ‘win’ for sensitizing even more of the population to the larger battle over the food system. By viewing GMO-labeling activism in this broader context, even those not directly involved drew from it; labeling initiatives brought more players to the game, and the size, diversity and momentum of the movement allowed for activism to snowball. Viewing the GMO battle in this way, as many activists did, casts its status more positively. For example:

...just as the biotech food genie is out of the bottle, the consumers’ wish to know genie is out of the bottle and ever-expanding. [3]

While the market strategies did not achieve immediate transformative outcomes, activists were nonetheless primarily empowered by the role they played in the broader battle for agricultural transformation.

## Discussion/conclusion

While both mandatory and voluntary GMO labeling can provide the tools to eat GMO-free, it would be difficult to characterize either market strategy as a movement success if it undermined more transformative efforts. Firstly, conceptualizing the case study to encompass the broader network of related organizations confirmed that the impacts of such mobilizations can extend well beyond the immediate initiators of an action and supports assessing ‘cases’ more comprehensively when evaluating the impacts of neoliberalized strategies.

Secondly, notwithstanding concerns raised over the NGMOP, neither activists nor the overall biotechnology movement appeared weakened by labeling strategies—when we cast their movement goals more broadly, as activists themselves did. Not only did GM activism not die, as evidenced by subsequent policy efforts, but activists as a whole remained committed to a cycle of contention around food that is steeped in regulatory skepticism and opposition to a corporate industrial model of agriculture where profit trumps all values. If anything, the apparent federal disregard of citizens’ wishes around GMOs—even when molded to neoliberal ideals of responsibilized citizen-consumers—only strengthened activists’ mistrust of its regulatory priorities and bolstered their activism.

Third, this study acts as a partial counter to concerns over neoliberal strategies’ impact on activists’ strategic abilities (Guthman 2008a, b). Most particularly with respect to the mandatory labeling efforts, activists unequivocally avoided the danger of neoliberalization that Harrison (2008) found when strategies were “severed from the broader fights for responsible and fair government protection” (2008, p. 1211). Moreover, in keeping with McClintock (2014), this study finds support for neoliberalization as both exemplifying “an actually existing neoliberalism” and a “simultaneous radical counter-movement” (2014, p. 148). Even neoliberal strategies that can lead to radical reform (such as a de facto GMO ban) can be transformative. Market strategies’ neoliberalizing impact on consumers remains a concern, however, which, if activists’ perception on this front were correct, bears consideration and raises an important area for further investigation. While market strategies can reify the primacy of the market, in a context of limited alternatives and a broad base of public support, their risk-benefit calculation weights differently.

Overall, the findings of this case study suggest that the characterization of strategies as neoliberalizing or not cannot be fairly assessed on ideological grounds, but by the extent of neoliberalization in their use and outcomes—that is, as ‘more’ or ‘less’ neoliberalized in a given context. In the wide-scale battle over the value priorities of the U.S. food system, these findings indicate that such strategies do not define the outcomes, but their use can have larger consequences for the course of future contestation.

Of the two strategies, mandatory labeling had the highest profile, and the impact of its market-based nature was the least problematic for the broader movement. Labeling was somewhat out of alignment with the stated goals of a number of activists, who felt forced to support the strategy over dwindling alternatives. As labeling can only be used to remove GMOs from the market, not improve their oversight, this strategy is a sloppy substitute for targeting regulatory shortcomings. In a context with more specific goals and other strategic options, this bears consideration. Whether consumer pressure could have triggered the withdrawal of GMOs from the market was not tested, and—given the shortcomings of the federal rules—never will be. No strategy guarantees success, however. Despite the defeat of preemption, activists remained savvy to context, highly strategic, and very motivated as forces of food system change.

With respect to the private GMO-free standard, the results suggest a greater concern with neoliberalization. Although the NGMOP was initiated with transformative goals, it struggled with its relationship to the market. While many hailed it as a means to express a nutritional and political perspective they were otherwise denied, concerns nonetheless abounded over its market priorities—which could distract well-intentioned consumers with products that had no reformative value—and its adverse impact on organic and small producers. The tension with organics, in particular, offered an important challenge to the potential for food system transformation. By acting in unintentional competition with another vehicle for change—wider-scale change, at that—the NGMOP hampered important efforts of the broader movement and created tensions within it. Although further case studies are necessary, this dynamic suggests that voluntary labels pose the greatest risk to movements, most particularly where credence claims can overlap.

## Abbreviations

AFM: alternative food movement
GM: genetically modified
GMO: genetically modified organism
NGMOP: Non-GMO Project
NBFDS: National Bioengineered Food Disclosure Standard

## Footnotes

1 Genetically Modified Organisms [GMOs] are organisms that have been genetically altered, such as through the introduction of genes from one species into another, creating transgenic organisms. They are also referred to as Genetically Engineered [GE], or more colloquially as biotechnology or biotech.

2 ‘GMO’ or ‘biotech’ activist is used here instead of ‘anti-GMO’ or ‘anti-biotech,’ in keeping with the wishes of a number of activists. This is in part because of the negative political stigma of being ‘anti-GMO’ (which is associated with more radical activities, such as tearing up GM corn fields) and in part because some object to the technologies’ regulatory framework more so than to the technologies themselves. The terms operate in contrast to ‘biotechnology proponents,’ who promote the technologies and often favour their deregulation.

3 It is not possible to identify these without identifying participants.

4 Ballot initiatives are citizen-initiated processes (which vary by state) that allow voters who collect sufficient signatures to put laws on the ballot for vote. Ballot initiatives are available in 26 U.S. states (Ballotpedia n.d.c).

5 For example, Connecticut required four other states, one bordering, and a minimum representative population of 20 million from regionally specified states for the law to take effect, while Maine required five states or a combined population of 20 million people (Arnold & Porter 2013).

6 As federal law is the supreme law of the land, it will supersede any conflicting state law. While courts disfavour preemption where the intent is ambiguous, the NBFDS contained two provisions expressly prohibiting “states and localities from enacting non-identical labelling laws” (Smith 2017: 19).

7 While GMO food labels are not in themselves ‘transformative,’ the use of these labels to de facto ban GMOs could be transformative in their impact on the U.S. pro-biotechnology paradigm, which facilitates many other aspects of the nation’s agribusiness. While ultimate outcomes are difficult to predict, and may not match with activist goals, the resulting instability could force changes such as the greater incorporation of citizen regulatory input in order to pre-empt future disruptions. As suggested by food regime analysis (see, for example, Friedmann 2009), instability provides opportunities for reconfiguration and can impact the global food regime, although this discussion is beyond the scope of this paper.

8 Claims that consumers cannot independently verify but must rely on provided information.

9 Herbicide associated with Monsanto’s “Roundup Ready” GM seeds.

## Declarations

### Declarations

Research was conducted with the ethical approval of the University of the Fraser Valley’s Human Research Ethics Board, Protocol #1074S-18. Informed consent was obtained from all individual participants included in the study.
